# The latest from the DMM team – 2026 edition

**DOI:** 10.1242/dmm.052843

**Published:** 2026-02-09

**Authors:** Rachel Hackett, E. Elizabeth Patton

**Affiliations:** ^1^ The Company of Biologists, Bidder Building, Station Road, Histon, Cambridge CB24 9LF, UK; ^2^ MRC Human Genetics Unit and CRUK Scotland Centre and Edinburgh Cancer Research, Institute of Genetics and Cancer, Western General Campus, The University of Edinburgh, Edinburgh EH4 2XU, UK

## Abstract

**Summary:** DMM outlines its achievements during 2025 and looks to the year ahead, with a thank you to our named peer reviewers for their vital input to the success of the journal.

## On the road with DMM

2025 was a busy year for DMM, the highlight being the Company of Biologists’ 100 anniversary celebrations (https://doi.org/10.1242/dmm.052792). We marked this important milestone with numerous activities throughout the year, including a unique scientific conference that took place in March. DMM played its part by hosting a fascinating and thought-provoking day-long strand on ‘Interdisciplinary approaches to combatting antimicrobial resistance’, organised by Katherine Duncan (Newcastle University, UK) and DMM Editorial Advisory Board member Serge Mostowy (London School of Hygiene & Tropical Medicine, UK) (see Mostowy and Duncan, 2025).

The next adventure for DMM will be in October 2026 in Ohio, USA, for our meeting on Innovative Preclinical Models for Pediatric Cancer Research. Advances in next-generation sequencing have enabled unprecedented cataloguing of genetic aberrations in tumours, but understanding how these genetic changes drive cellular transformation and how they can be effectively targeted for therapy, will require multidisciplinary collaboration and preclinical models that are truly representative of the *in vivo* environment. Organisers Jim Amatruda, Mimi Bandopadhayay, Ana Banito and Elaine Mardis have lined up an outstanding group of speakers, who will discuss new models and technologies that have the potential to transform our understanding of paediatric cancers.

November 2026 sees us in East Sussex, UK, for a Workshop on Integrating Multi-Modal, Multi-Scale Models of Cardiovascular Disease Mechanisms, with organisers Jennifer Davis, Christine Mummery and Beth Pruitt. This smaller event will focus on the novel models and innovations that will underpin important scientific advances in cardiovascular disease research. We can't wait!

## Special issues with integrity

DMM has been publishing special issues on topics of interest to our readers since 2014. However, such a practice is now under particular scrutiny from the community as a result of concerns regarding research integrity (Crosetto et al., 2026). At DMM, special issue themes and their Guest Editors are identified through discussions with members of the DMM Editor team and/or the Editorial Advisory Board. Invited Guest Editors are responsible for outlining the scope of the special issue, and also identifying topics and authors for the review-type articles that DMM publishes; the entire process is carefully overseen by the journal's Editorial Office and the Editor-in-Chief retains overall responsibility for the content. Peer review of submitted Research Articles is handled by the existing team of DMM Editors and all standard publication ethics policies of the journal apply. Editorial decisions for manuscripts for which Guest Editors are authors are made entirely independently of the Guest Editors. With these policies in place, we strongly believe that readers can be confident in the integrity of our special issue content.

Our 2025 Special Issue, Infectious Disease: Evolution, Mechanism and Global Health – guest edited by Judy Allen, Sumana Sanyal, David Tobin and Russell Vance, is now complete. As infectious diseases continue to challenge health organisations worldwide, this latest special issue seeks to contribute to the vital continuing conversation between basic scientists, clinical researchers and clinicians. Read the introductory Editorial to find out more about the articles published in that special issue (Hackett et al., 2025a). And remember DMM continues to welcome submissions on these topics in the future.

June 2026 will see the publication of DMM's latest Special Issue – *In Vitro* Models of Human Disease to Inform Mechanism and Drug Discovery. Advances in the development and application of human stem-cell-based models and tools are critical for disease research and therapeutic development. Edited by DMM Editor Vivian Li, alongside Guest Editors Austin Smith and Joseph Wu, it will include our usual excellent selection of Reviews and Research Articles, which will describe the application of complex human cellular models, such as organoids, assembloids, tumour organoids, organs-on-a-chip and gastropods, to inform disease mechanisms and drive the discovery of novel therapeutics.

## Supporting the research and researchers of tomorrow

DMM will continue its commitment to supporting early-career researchers (ECRs) (Hackett, 2025a). Our ‘First Person’ interviews during 2025 featured over 40 ECR authors of papers published in the journal, highlighting not only their excellent research but also their dedication to improving human health. More than 180 ECRs were the deserved recipients of our Conference Travel Grants and 30 more received Travelling Fellowships. Respectively, these offer financial support to ECRs wanting to attend conferences, workshops and training courses that relate to the scope of DMM, and funds for collaborative visits to other laboratories. In addition, we will soon be short-listing candidates for our Outstanding Paper Prize, which is awarded to the first author(s) of the papers that are judged by DMM's Editors to be the most-outstanding contributions to the journal that year (Hackett 2025b).

## DMM's expert teams

DMM is lucky enough to have an outstanding Editorial Advisory Board. The board has an important role for the journal, providing invaluable expertise and advice to Editors on submitted manuscripts, and to the Editor-in-Chief regards journal strategy and development.

We are grateful to those Board members who stepped down at the end of their term this past year for their valued service and dedication to the journal over the years: Frances Balkwill (Barts Cancer Institute, UK), Dirk Bohmann (University of Rochester Medical Center, USA), Nancy M. Bonini (University of Pennsylvania, USA), Robert Cardiff (University of California Davis, USA), Sabine P. Cordes (Lunenfeld-Tanenbaum Research Institute, Canada), Elaine Dzierzak (The University of Edinburgh, UK), Jonathan A. Epstein (Perelman School of Medicine, USA), Elizabeth Fisher (University College London, UK), Yasuyuki Fujita (Kyoto University Medical School, Japan), Aron M. Geurts (Medical College of Wisconsin, USA), Brigid Hogan (Duke University, USA), Raghu Kalluri (MD Anderson Cancer Center, USA), Richard I. Morimoto (Northwestern University, USA), Stefano Piccolo (University of Padua, Italy) and Chad A. Shaw (Baylor College of Medicine, USA). We will be inviting new members to join the team throughout 2026.

DMM welcomed Shinya Yamamoto (Baylor College of Medicine, USA) to the team of research-active Editors who handle research submissions to DMM. Shinya works on the integration of *Drosophila* genetics and human genomics, new disease gene discovery, *Drosophila* technology and resource development, cell-cell communication in development and disease, and bioinformatic tool development. We also appointed Satdarshan (Paul) Singh Monga (University of Pittsburgh, USA). His research focuses on understanding the cellular and molecular mechanisms of liver development, regeneration and cancer. DMM is delighted to have such outstanding new Editors join our team; you'll find out more about them in a future editorial.

## Our thanks to the whole DMM community

DMM would also like to take the opportunity, as we do at the start of every year, to thank our reviewers for their time, expertise and dedication. The names of all our 2025 reviewers, including the co-reviewers, are listed in Box 1. We also thank those reviewers of articles transferred to DMM from Review Commons.

We thank our Editorial Advisory Board: your time, expertise and thoughtful input are vital to the journal's strategy and integrity.

We are particularly grateful to our Editors, who spend countless hours assessing manuscripts (see Box 2 for our most-read and -cited articles during 2025), making editorial decisions and working to maintain DMM's reputation. We know that your DMM work draws you from your own research, teaching and home lives, and appreciate your expertise and dedication.

To our authors, we are delighted you trusted us to publish your work and hope you found it to be a positive experience (Box 3). DMM is part of The Company of Biologists family; by publishing in DMM you are supporting a not-for-profit charity run by scientists for scientists.

We are hugely grateful to all who support us now and, we hope, in the future.

Box 2. Highlighted articles 2025 **Most read during 2025**

James Chung, Julia Pierce, Craig Franklin, Rachel M. Olson, Alan R. Morrison, James Amos-Landgraf

Translating animal models of SARS-CoV-2 infection to vascular, neurological and gastrointestinal manifestations of COVID-19

Victor S. Tapia, Sarah E. Withers, Ran Zhou, Abigail Bennington, Christopher Hoyle, Frances Hedley, Adam El Khouja, Nadim Luka, Marco Massimo, Siobhan Crilly, Katherine R. Long, Catherine B. Lawrence, Paul R. Kasher

The role of 25-hydroxycholesterol in the pathophysiology of brain vessel dysfunction associated with infection and cholesterol dysregulation

**Most cited during 2025**

Priya D. Gopal Krishnan, Wen Xing Lee, Kah Yong Goh, Sze Mun Choy, Lewin Raymarc Roldan Turqueza, Zhuo Han Lim, Hong-Wen Tang

Transcriptional regulation of autophagy in skeletal muscle stem cells

Meri Uusi-Mäkelä, Sanna-Kaisa Emilia Harjula, Maiju Junno, Alina Sillanpää, Reetta Nätkin, Mirja Tellervo Niskanen, Anni Karoliina Saralahti, Matti Nykter, Mika Rämet

The inflammasome adaptor *pycard* is essential for immunity against *Mycobacterium marinum* infection in adult zebrafish

Data from Impact Vizor.

Box 3. Feedback from our community of published authors I very much appreciated the process of publishing our latest paper in DMM. I found the submission easy, the reviewers’ comments and requests were fair and helpful, and the production team very efficient. Overall, it was a very enjoyable and positive experience. I will definitely keep DMM in mind as a journal of choice when we have a new manuscript that could be in scope.

I was thrilled to have our image on the front cover, what an honour! It was a great experience publishing in DMM, I can tell you it was a much smoother and less painful process than other publishers.

I received very constructive, thorough, and directed reviewer feedback on our submission – the review was one of the highest quality I've had. There was a considerable delay in getting the second review back, but the quality of the review was worth the wait. We will absolutely keep DMM in mind for our next submission.

We appreciate the excellent work by both the scientific editor and production editor throughout the publication process of our manuscript. Special thanks to the in-house production team for their careful attention to detail in enhancing the final version – this has been the best publication experience I've had. We will definitely consider submitting our future work to DMM.

Box 1. Reviewers for Disease Models & Mechanisms during 2025 To ensure that DMM publishes rigorously conducted high-quality research, we are dependent on the time and expertise of our peer reviewers. We take this opportunity to thank all the reviewers and co-reviewers (and reviewers of articles transferred to DMM from Review Commons), who dedicated their time to contributing thoughtful feedback that advances science and how it is communicated.

Omar Abdel-Wahab, Memorial Sloan Kettering Cancer Center, USA

Adeleye Afolayan, Medical College of Wisconsin, USA

Devansh Agarwal, University California San Diego, USA

Mir Hilal Ahmed, Jawaharlal Nehru University, India

Guru Ajay, Marwadi University, India

Nino Akkerman, Hubrecht Institute for Developmental Biology, The Netherlands

Kimberly A. Aldinger, Seattle Children's Research Institute, USA

Matthew Alexander, University of Alabama at Birmingham, USA

Dominique Alfandari, University of Massachusetts Amherst, USA

Carlos Almeciga-Diaz, Pontificia Universidad Javeriana, Colombia

James Amatruda, Children's Hospital Los Angeles, USA

Lutong An, CRUK Manchester, UK

Corina Anastasaki, Washington University in St Louis, USA

Kanae Ando, Tokyo Metropolitan University Graduate School of Science, Japan

Eran Andrechek, Michagan State University, USA

Daria Andreeva, University of Karlsrheu, Canada

Jonathan Andrews, Baylor College of Medicine, USA

Rifdat Aoidi, The Crick Institute, London, UK

Vikas Arige, University of Rochester, USA

Ami Aronheim, Technion Israel Institute of Technology, Israel

Ignacio Babiloni Chust, University of Trento, Italy

Andy Badrock, The University of Edinburgh, UK

Baskar Bakthavachalu, Indian Institute of Technology, India

Scott Baraban, University of California, San Francisco, USA

Mirella Barboni, Semmelweis University, Hungary

Vanessa Barone, Stanford University, USA

Pamela Barraza-Flores, Boston Children's Hospital, USA

William Barrell, King's College London, UK

Peter Barr-Gillespie, Oregon Health and Science University, USA

Sina Bartfeld, Technische Universitat Berlin, Germany

Enrico Baruffini, University of Parma, Italy

Taylor Barwell, Children's Hospital of Eastern Ontario Research Institute, Canada

Peter Bass, Drexel University, USA

M. Albert Basson, King's College London, UK

Anirban Basu, National Brain Research Centre, India

Alexandra Belayew, University of Mons, Belgium

Samuel Bertrand, University of Oregon, USA

Joep Beumer, Roche Institute of Human Biology, Switzerland

Jelena Bezbradica, University of Oxford, UK

Anita Bhattacharyya, University of Wisconsin-Madison, USA

David Bilder, University of California-Berkeley, USA

Karen Blyth, CRUK Scotland Institute, UK

Jeffrey Boatright, Emory University School of Medicine, USA

Enrica Boda, UNITO: Universita degli Studi di Torino, Italy

Nancy Bonini, University of Pennsylvania, USA

Markus Bosmann, Boston University, USA

Tamara Boto, University of Bristol, UK

Sevda Boyanova, University College London, UK

Tomas Brdicka, Czech Academy of Sciences, Czech Republic

David Breslow, Yale University, USA

Nika Breznik, University of Ljubljana, Slovenia

Andreas Brodehl, Heart and Diabetes Center NRW, Germany

Ann-Christin Brorsson, Linkopings Universitet, Sweden

Michael Bround, Baylor College of Medicine, USA

Hannah Brunsdon, University of Edinburgh Western General Hospital, UK

Ruslana Bryk, Weill Cornell Medicine, USA

Lori Buhlman, Midwestern University, USA

Margret Bülow, University of Dusseldorf, Germany

Alexa Burger, University of Colorado, USA

Claudio Bussi, Nanyang Technological University, Singapore

Brian Calvi, Indiana University Bloomington, USA

Alex Cammack, University College London, UK

Maria Capovilla, Institut de Pharmacologie Moleculaire et Cellulaire, CNRS, France

Mathias Carl, University of Trento, Italy

Leo Carlin, CRUK Scotland Institute, UK

Richard L. Carpenter, Indiana University School of Medicine, USA

Trae Carroll, University of Rochester, USA

Sergio Casas, Unidad de Modelos de Enfermedades Humana, Spain

Elena Cattaneo, University of Milano, Italy

Maria Chahrour, The University of Texas Southwestern Medical Center, USA

Yang Chai, University of Southern California, USA

Sunandan Chakrabarti, Indiana University, USA

Sayan Chakraborty, Roswell Park Comprehensive Cancer Center, USA

Andrew Chan, The Chinese University of Hong Kong School of Biomedical Sciences, Hong Kong

Danny Chan, The University of Hong Kong, Hong Kong

Vijayendran Chandran, University of Florida, USA

Hsiao Tuan Chao, Baylor College of Medicine, USA

Haiyang Chen, Sichuan University, China

Jing Chen, China Three Gorges University, China

Ya Chen, Case Western University, USA

Yi-Wen Chen, Children's Research Institute, USA

Andy Cheng, University of Alberta, USA

Mein Chew, Cambridge, UK

Adam Chicco, Colorado State University, USA

Budhaditya Chowdhury, Bucknell University, USA

David Clancy, Lancaster University, UK

Lucy Clarke, University of Glasgow, UK

Julie Cohen, Tulane University, USA

Justin Cohen, Yale University School of Medicine, USA

Holly Colognato, Stony Brook University, USA

Christopher Colwell, UCLA, USA

Julia Cordero, University of Glasgow, UK

D.D.W. Cornelison, University of Missouri, USA

Andrew Cox, Peter MacCallum Cancer Centre, Australia

Richard Cripps, San Diego State University, USA

Milena Damulewicz, Jagiellonian University, Poland

Robin Dart, King's College London, UK

Della David, Babraham Institute, UK

Ben Davies, Francis Crick Institute, UK

Maria De Grandis, Etablissement Francais du Sang, France

Jose de la Pompa, Centro Nacional de Investigaciones Cardiovasculares Carlos III, Spain

Annamaria De Luca, University of Bari, Italy

Carmen del Rio, Spanish National Research Council, Spain

Hansong Deng, Tongji University, China

Qing Deng, Purdue University, USA

Jorn Dengjel, Universite de Fribourg, Germany

Nicole Desmet, Michigan State University, USA

Francesca Di Cara, Dalhousie University, Canada

Katja Dierking, University of Kiel Faculty of Medicine, Germany

Susanne Dietrich, University of Portsmouth, UK

Miriam Domowicz, University of Chicago, USA

Rajashekar Donaka, University of Colarado, USA

Anca Dorhoi, Friedrich Loeffler Institut, Germany

James Dowling, Hospital for Sick Children, Canada

Rahul Dubey, Jawaharlal Nehru University, India

Carrie Duckworth, The University of Liverpool, UK

Sally Dunwoodie, Victor Chang Cardiac Research Institute, Australia

Luc Dupuis, INSERM, France

Delphine Duteil, Université de Strasbourg, France

Glorida Echeverria, Baylor College of Medicine, USA

Randall Eck, University of Washington, USA

Kirsten Edepli, New York University Abu Dhabi, United Arab Emirates

George Eisenhoffer, The University of Texas MD Anderson Cancer Center, USA

Marc Ekker, University of Ottawa, Canada

Elie El Agha, Justus Liebig University Giessen, Germany

Kornelia Ellwanger, University of Tübingen, Germany

Heba Elsallab, University College London, UK

James Ervasti, University of Minnesota, USA

Cristina Espinosa-Diez, Wayne State University, USA

Carlos Estella, Centro de Biologia Molecular, Spain

Changfa Fa, National Institutes for Food and Drug Control, China

Colin Farquharson, The University of Edinburgh, UK

Sina Fatehi Someeh, University of Toronto, Canada

Philippa Francis-West, King's College London, UK

Sabine Fuhrmann, Vanderbilt University Medical Center, USA

Yuka Fujita, Riken, Japan

Eliska Furlong, Perth Children's Hospital, Australia

Maura Galimberti, Murdoch Children's Research Institute, Australia

Francesco Galli, University of Perugia, Italy

Subramaniam Ganesh, Indian Institute of Technology, India

Victoria Garside, The University of Melbourne - Parkville Campus, Australia

Susanne Gaul, University of Leipzig Faculty of Medicine, Germany

Kinga Gawel, University of Oslo, Norway

Samit Ghosh, University of Pittsburgh, USA

Stephanie Goldstein, University of Utah, USA

Christian Gonzalez, Texas A&M University of Biology, USA

Mercedes Gonzalez-Juarrero, Colorado State University, USA

Jaclyn M. Goodrich, University of Michigan School of Public Health, USA

Yogesh Goyal, Northwestern University Feinberg School of Medicine, USA

Erin Greaves, University of Warwick, UK

Nicholas Greene, University of Arkansas, USA

James Griffin, University of Exeter, UK

Agi Grigoriadis, King's College London, USA

Daniel Grimes, University of Oregon, USA

Yebo Gu, Tongji University School of Medicine, Japan

Zhongze Gu, Southeast University, China

Rachel Guest, University of Edinburgh, UK

Eric Guisbert, University of Nebraska Omaha, USA

Asiya G, Duke University School of Medicine, USA

Tripti Gupta, National Institutes of Health, USA

Sonam Gurung, University College London, UK

Jennifer Gutzman, University of Wisconsin-Milwaukee, USA

Dafni Hadjieconomou, Paris Brain Institute: Institut du Cerveau, France

Daniel Ham, University of Basel, Switzerland

Hiroshi Hamada, RIKEN Noshinkei Kagaku Kenkyu Center, Japan

Kihoon Han, Korea University, Republic of Korea

Malene Hansen, Buck Institute for Research on Aging, USA

Yutaka Harita, The University of Tokyo, Japan

Patrick Harrison, University College Cork, Ireland

Silvia Hayer, Medical University of Vienna, Austria

Claire Healy, Trinity College Dublin, Ireland

Christopher Heier, Virginia Commonwealth University, USA

Ilka Heinemann, University of Western Ontario: Western University, Canada

Katrin Henke, Emory University School of Medicine, USA

Yann Herault, Institut de Genetique et de Biologie Moleculaire et Cellulaire, CNRS, France

Tobias Hermle, University of Freiburg, Germany

Gary Hime, University of Melbourne, Australia

Nina Himmerkus, Christian-Albrechts-Universitat zu Kiel, Germany

Eric P. Hoffman, Binghamton University, USA

Peter Hohenstein, Leids Universitair Medisch Centrum, The Netherlands

Sandra Holley, University of California Los Angeles, USA

Gregory Holmes, Icahn School of Medicine at Mount Sinai, USA

Martin Hoogduijn, Erasmus Medical Centre, The Netherlands

Rita Horvath, University of Cambridge, UK

Negar Hosseini, University of Southern California, USA

Shushu Huang, Yale University, USA

Peng Huang, University of Calgary, Canada

Xun Huang, Wuhan University, China

Sandrine Humbert, Hôpital Universitaire Pitie Salpêtrière, France

David Humphreys, Victor Chang Cardiac Research Institute, Australia

Robert Hynds, University College London, UK

James Iatridis, Icahn School of Medicine at Mount Sinai, USA

Narthana Ilenkovan, Beatson Institute for Cancer Research, UK

Svenja Illien-Junger, Emory University School of Medicine, USA

Gareth Inman, CRUK Beatson Institute, UK

Adrian Isaacs, UCL Institute of Neurology, UK

Albert Isaacs, Nationwide Children's Hospital, USA

Sachiko Iseki, Tokyo Medical and Dental University, Japan

Kinya Ishikawa, Tokyo Medical and Dental University: Tokyo Ika Shika Daigaku, Japan

Tohru Ishitani, Osaka University Faculty of Medicine Graduate School of Medicine, Japan

Evgueni Ivakine, Hospital for Sick Children, Toronto, Canada

Takeshi Iwata, NHO Tokyo Medical Center, Japan

Rob Jackson, Tufts University, Sackler School of Graduate Biomedical Sciences, USA

Timothy Jacobsen, School of Medicine at Mount Sinai, USA

Pudur Jagadeeswaran, University of North Texas, USA

Archana Jayaraman, Boston University, USA

Xinming Jia, Tongji University School of Medicine, China

Veronica Jimenez, Universidad Autónoma de Barcelona, SPAIN

Aaron Johnson, Washington University at St Louis, USA

Mohit Kumar Jolly, Indian Institute of Science, India

Spencer Jones, Radboudumc, The Netherlands

Mark Kahn, University of Pennsylvania, USA

Manjula Kalia, Regional Centre for Biotechnology, India

Alexandros Kanellopoulos, DSM Research and Development, Switzerland

Matthia A. Karreman, Universitatsklinikum Heidelberg, Germany

Shingo Kato, Yokohama City University Urafane Hospital, Japan

Vladimir Kefalov, University of California Irvine, USA

Ines Kelleher Lopez, CBM, Spain

David Kent, University of York, UK

Brennen Keuchel, Buck Institute, USA

Zoha Kibar, University of Montreal, Canada

Sang-guen Kim, Kyonggi University, South Korea

Sung-Eun Kim, The University of Texas at Austin, Dell Medical School, USA

Jacqueline M. Kimmey, University of California Santa Cruz, USA

Kirill Kiselyov, University of Pittsburgh, USA

Robert Knight, King's College London, UK

Klaus-Peter Knobeloch, University of Freiburg Faculty of Medicine, Germany

Dwight Koeberl, Duke University, USA

Anna Konopka, Flinders University, Australia

Nataša Kopitar-Jerala, Jozef Stefan Institute, Slovenia

Sirin Korulu Koc, Tallinn University, Estonia

Brian Kraemer, University of Washington, USA

Balaji Krishnan, The University of Texas Medical Branch at Galveston, USA

Michael Kuehl, Universitat Ulm, Germany

Jeffrey Kuerbitz, Baylor College of Medicine, USA

Anirban Kundu, Arizona State University, USA

Christian Kupatt, Technical University Munich, Germany

Gopal Kushawah, Stowers Institute for Medical Research, USA

Young Kwon, University of Washington Seattle Campus, USA

Gyanu Lamichhane, Johns Hopkins University, USA

Eva Lana-Elola, Francis Crick Institute, UK

Konrad Lang, University of Freiburg, Germany

Paul G. LaPointe, University of Alberta, USA

David Largaespada, University of Minnesota Masonic Cancer Center, USA

Tal Laviv, Tel Aviv University, Israel

Anna-Lisa Lawrence, Tufts University Medical School, USA

Laurence Legeai-Mallet, INSERM, France

Imre Lengyel, Queen's University Belfast, UK

Dorothy Lerit, Emory University School of Medicine, USA

Tylor Lewis, University of Alabama at Birmingham, USA

Yun Li, University of Toronto, Canada

Christine Li, The City College of New York, USA

Shuangxi Li, Shandong University, China

Heiko Lickert, Helmholtz Zentrum Munich, Germany

Angus Lindsay, University of Canterbury, New Zealand

Qinglan Ling, University of Massachusetts Chan Medical School, USA

Nichole Link, University of Utah, USA

Zhiqiang Liu, Shandong First Medical University, China

Andrew C. Liu, University of Florida College of Medicine, USA

Shuangxin Liu, Guangdong Provincial People's Hospital of Southern Medical University, China

Yan Liu, Nanjing Medical University, China

Hannah Long, University of Edinburgh, UK

Susana S. Lopes, University of Lisbon Faculty of Sciences, Portugal

Manuel Lopez, Biomarin Pharmaceutical Inc, USA

Jose Lopez-Escamez, The University of Sydney, Australia

Ben Lovely, University of Louisville School of Medicine, USA

Martin P. Lowe, Manchester University, UK

Emilia Luca, Sunnybrook and Women's College Health Sciences Centre, Canada

Kathy O. Lui, Chinese University of Hong Kong, Hong Kong

Bryan W Luikart, University of Alabama Hospital, USA

Amaia Lujambio, Icahn School of Medicine at Mount Sinai, USA

Kaiyue Ma, Shanghai Jiao Tong University, China

Xianjue Ma, Westlake University, China

Lesley MacNeil, McMaster University, Canada

Tomoko Makishima, University of Texas Medical Branch, USA

Brigitte Malgrange, University of Liege, Belgium

Ravi Manjithaya, Jawaharlal Nehru Institute of Advanced Scientific Research, India

Zoe Mann, King's College London, UK

Maria Marchese, IRCCS Fondazione Stella Maris, Italy

John Mariadason, Olivia Newton-John Cancer Wellness & Research Centre, Australia

Mir Farzin Mashreghi, Deutsches Rheuma-Forschungszentrum Berlin, Germany

Molly Matty, University of Portland, USA

Lisa Maves, Seattle Children's Research Institute, USA

Simone Mayer, Karlsruhe Institute of Technology, Germany

Jessica McCann, Duke University, USA

Heather McCauley, University of North Carolina, USA

Barry McColl, University of Edinburgh, UK

Stephen McGowan, The University of Iowa, USA

Serge McGraw, University of Montreal, Canada

Zach McLean, Massachusetts General Hospital, USA

James McNamara, Murdoch Children's Research Institute, Australia

Roly Megaw, University of Edinburgh Western General Hospital, UK

Annemarie Meijer, Leiden University, The Netherlands

Sigolene Meilhac, Institut Pasteur, France

Valeria Melis, University of Aberdeen, UK

Diane Merry, Thomas Jefferson University, USA

Alison Michie, University of Glasgow, UK

Pleasantine Mill, The University of Edinburgh, UK

Berge Minassian, UT Southwestern, USA

Laxmi Mishra, Norwich Research Park, UK

Pramod Mistry, Yale University School of Medicine, USA

Diana Mitchell, University of Idaho, USA

Cristina Molnar, Barcelona Institute for Science and Technology, Spain

Zoltan Molnar, University of Oxford, UK

Amal Mondal, Jawaharlal Nehru University, India

Joan Montaner, Universidad de Sevilla, Spain

Sally Moody, George Washington University Medical Center, USA

Orson Moritz, University of British Columbia, USA

Serge Mostowy, London School of Hygiene and Tropical Medicine, UK

Matthew Moulton, Texas A&M University, USA

Darrell David Mousseau, University of Saskatchewan, Canada

Henricus Mutsaers, Aarhus University, Denmark

Sonal Nagarkar Jaiswal, Centre for Cellular and Molecular Biology CSIR, India

Shiny Nair, Yale University, USA

Yuichiro Nakajima, University of Tokyo, Japan

Soumya Navneet, Medical University of South Carolina, USA

Keith Nehrke, University of Rochester, USA

Lukas Ne, University of Lausanne, Switzerland

Timothy Nguyen, Iowa Graduate College, USA

Teresa Niccoli, University College London, UK

Peter Noakes, The University of Queensland, Australia

Fanny Nobilleau, Universite Montreal, Canada

Satoru Noguchi, Kokuritsu Kenkyu Kaihatsu Hojin Seishin Shinkei Iryo, Japan

Monika Nowak-Imialek, Technical University of Munich Hospital Rechts der Isar, Germany

Fumiaki Obata, RIKEN Center for Biosystems Dynamics Research, Japan

Amaia Ochandorena Sa, Institut Pasteur, France

Stefan Oehlers, Agency for Science Technology and Research, Singapore

Tomoko Ohyama, McGill University, Canada

Mario Ollero, Universite Paris-Est Creteil Val de Marne, France

Kiel Ormerod, Middle Tennessee State University, USA

Tala Ortiz, UF Center for Neurogenetics, USA

Antonio Pagán, Stanford University School of Medicine, USA

Alexandre Paix, Max Planck Institute for Developmental Biology, Germany

Dashika Palipana, Victor Chang Cardiac Research Institute, Australia

Duojia Pan, University of Texas Southwestern Medical Center, USA

Vanessa Parietti, CRUK Manchester, UK

Paul Park, Case Western Reserve University, USA

Suhel Parvez, Jamia Hamdard, India

E. Elizabeth Patton, The University of Edinburgh, UK

Henry Paulson, University of Michigan, USA

Neal Peachey, Cleveland Clinic, USA

Claire Pearson, University of Oxford, UK

Eric Peterman, University of Washington, USA

Lucia Poggi, University of Trento, Italy

Angelo Poletti, University of Milan, Italy

Steve Pollard, University of Edinburgh, UK

Manuela Priolo, SSD Genetica Medica, Portugal

Rituraj Purohit, CSIR-Institute of Himalayan Bioresource Technology, India

Sonja Pyott, University Medical Centre Groningen, The Netherlands

Junbin Qian, Zhejiang University, China

Giorgia Quadrato, University of Southern California, USA

Soundharya Ramu, CSB Lab Indian Institute of Science, India

Thomas Raabe, Julius-Maximilians-Universitat Wurzburg, Germany

Stuart Ralston, University of Edinburgh Western General Hospital, UK

Owen Randlett, MeLiS Institute, France

Laura Ranum, University of Florida, USA

Jeffrey Rasmussen, University of Washington, USA

J Arjuna Ratnayaka, University of Southampton, UK

Emma Rawlins, University of Cambridge, UK

John Rawls, Duke University School of Medicine, USA

Jan Rehwinkel, University of Oxford, UK

Lawrence Reiter, Tulane University, USA

Leah Reznikov, University of Florida, USA

David Rice, University of Helsinki, Finland

Joy Richman, The University of British Columbia Faculty of Dentistry, Canada

Edward Roberts, Beatson Instititute for Cancer Research, UK

Alexander Robling, Indiana University School of Medicine, USA

Kyle Rohde, University of Central Florida, USA

Palle Rohde, Aalborg Universitet, Denmark

Bärbel Rohrer, Medical University of South Carolina, USA

Camilla Roselli, Trinity College Dublin, Ireland

Emily Rosowski, Clemson University College of Science, USA

Emil Rudolf, Univerzita Karlova, Czech Republic

Holger Russ, University of Florida, USA

Jean-Pierre Saint-Jeannet, New York University College of Dentistry, USA

Hidetoshi Sakurai, Kyoto University: Kyoto Daigaku, Japan

Eric Samarut, University of Montreal, Canada

Yuya Sanaki, University of Tsukuba Japan

Owen Sansom, Cancer Research UK Scotland Institute, UK

Filippo Santorelli, IRCCS, Italy

Mitsuru Sasaki Honda, Kyoto University, Japan

Smita Saxena, University of Missouri, USA

Elena Scarpa, Cambridge University, UK

Christian Schaaf, Heidelberg University, Germany

Hein Schepers, University Medical Centre Groningen, The Netherlands

Miriam Schmidts, Albert-Ludwigs-Universitat Freiburg, Germany

Sylvie Schneider-Manoury, Sorbonne Universite, France

Frauke Seemann, Texas A&M University – Corpus Christi, USA

Joachim Seemann, UT Southwestern Medical Center, USA

Bijoya Sen, Buck Institute, USA

Ines Serra, Erasmus Medical Centre, The Netherlands

Volkan Seyrantepe, Izmir Institute of Technology, Germany

George Sflomos, EPFL: Ecole Polytechnique Federale de Lausanne, Switzerland

Veeral Shah, Cincinnati Children's Hospital Medical Center, USA

Shivangi Sharma, University of Manitoba, USA

Timothy David Shaw, Queen's University Belfast, UK

John Shern, National Cancer Institute Center for Cancer Research, USA

Celia Shiau, The University of North Carolina at Chapel Hill School of Medicine, USA

Monika Shla, Indian Institute of Technology, India

Andrew Simmonds, University of Alberta Faculty of Medicine & Dentistry, Canada

Christophe Sirac, Limoges University, France

Mikael Skurnik, Helsingin Yliopisto Laaketieteellinen tiedekunta, Finland

Paulien H. Smeele, Universita degli Studi di Milano, Italy

Clare Smith, Duke University School of Medicine, USA

Charlie Softley-Brown, Keele University, UK

Khalid Sossey-Alaoui, Metrohealth Medical Center, USA

Katherine Staines, University of Brighton, UK

Viktoriya Stancheva, University of Oxford, UK

Josefa Steinhauer, Yeshiva University, USA

Rolf Stottmann, The Ohio State University, USA

Atsushi Sugie, Niigata University, Japan

Alyson Sujkowski, Wayne State University-Detroit, USA

Mark Sullivan, Harvard University Chan School of Public Health, USA

Isaac Kirubakaran Sundar, University of Kansas Medical Center, USA

Nana Sunn, Victor Chang Cardiac Research Institute, Australia

Sheyum Syed, University of Miami, USA

Mazazumi Tada, University College London, UK

Tamara Tal, Helmholtz-Centre for Environmental Research - UFZ, Germany

Vikram Tallapragada, Victor Chang Cardiac Research Institute, Australia

Michelle Tallquist, University of Hawaii, USA

Li Xuan Tan, University of Health and Rehabilitation Sciences, China

Shumin Tan, Tufts University School of Medicine, USA

Alan Tang, University of Pennysylvania, USA

Wucheng Tao, Fujian Medical University, China

Michel C. Tchan, Westmead Hospital, Australia

Nicholas Thomas, University of Kentucky, USA

Yannick Throm, Heidelberg University, Germany

Aiguo Tian, Tulane University of Louisiana, USA

David Ting, Massachusetts General Hospital, USA

Amit Tirosh, Sheba Cancer Center and Institute of Oncology, Israel

David Tobin, Duke University School of Medicine, USA

Sokol Todi, Wayne State University School of Medicine, USA

Shruti Tophkhane, University of Minnesota School of Dentistry, USA

Katarina Podkrajsek, University of Ljubljana, Slovenia

Carol Trempus, National Institute of Environmental Health Sciences, USA

Barbara Triggs-Raine, University of Manitoba, Canada

Stella Tsirka, Stony Brook University, USA

Wei-Ling Tsou, Wayne State University, USA

Yusuf Tutar, Recep Tayyip Erdogan University, Turkey

Victor Tybulewicz, The Francis Crick Institute, UK

Thomas Vaccari, Universita degli Studi di Milano, Italy

Eric Van Otterloo, University of Iowa, USA

Hugo Vankelcom, Katholieke Universiteit Leuven, Belgium

A. Catalina Velez-Ortega, University of Kentucky, USA

Charles Venditti, National Institutes of Health, USA

Siddharth Ravi Venkatesh, The Babraham Institute, UK

Esther Verheyen, Simon Fraser University, Canada

Hilary Vernon, Johns Hopkins University School of Medicine, USA

Amaya Viros, Cancer Research UK Manchester Institute, UK

Randi Vita, La Jolla Institute for Immunology, USA

Veronique Vitart, University of Edinburgh, UK

Pierre-Yves von der Weid, University of Calgary, Canada

Alex von Kriegsheim, University of Edinburgh, UK

Fernando Vonhoff, University of Maryland Baltimore County, USA

Natalia Vydra, Curie Memorial Cancer Center, Poland

Karl Wahlin, University of California San Diego, USA

Amy Walker, UMASS Medical School, USA

Maggie C. Walter, Friedrich-Baur-Institute, LMU Munich, Germany

Bo Wang, West China Hospital of Sichuan University, China

Raymond Wang, University of California Irvine, USA

Kirk Wangesteen, Mayo Clinic College of Medicine and Science, USA

Yuli Watanabe, INSERM, France

Dominic Wells, Royal Vetinary College, UK

Theodore Wensel, Baylor College of Medicine, USA

Nicole West, University of Wisconsin-Madison, USA

Robert Wheeler, University of Maine System, USA

Mary Whitman, Harvard Medical School, USA

Jonathan Whittamore, The University of Texas Southwestern Medical Center, USA

Alex Whitworth, University of Cambridge, UK

Adrienne Widener, University of Florida, USA

Wiesława Widłak, Maria Sklodowska-Curie National Research Institute of Oncology Gliwice Branch, Poland

Simon Wilkinson, Edinburgh Cancer Research Centre, UK

Alice Wood, Lancaster University, UK

Kathryn Wright, London School of Hygiene and Tropical Medicine, UK

Tongbin Wu, Masonic Medical Research Institute, USA

Anthony J. Wynshaw-Boris, Case Western Reserve University School of Medicine, USA

Stephanie Xie, University of Toronto, Canada

Ranjie Xu, Purdue University, USA

Xiaolei Xu, Mayo Clinic College of Medicine Learning Resource Center, USA

Rui Yang, Xi'an Jiaotong University, China

Yujiao Yang, Yale University, USA

Dingzi Yin, Mayo Clinic, USA

Toshinori Yoshida, Tokyo University of Agriculture and Technology, Japan

Li-Ru You, National Yang Ming Chiao Tung University, Taiwan

Stephane Zaffran, Aix-Marseille University, France

Gaihua Zhang, Hunan Normal University, China

Ke Zhang, Shenzhen Bay Laboratory, China

Qi Zhang, Yale School of Medicine, USA

Shuo Zheng, Virginia Commonwealth University, USA

Yi Zhou, Florida State University College of Medicine, USA

ZhaoLan Zhou, University of Pennsylvania, USA

Alan Zhu, Peking University, China

Hao Zhu, The University of Texas Southwestern Medical Center, USA

Shougang Zhuang, The Warren Alpert Medical School, USA

Christiane Zweier, University of Bern, Switzerland
